# Modeling Signal Propagation Mechanisms and Ligand-Based Conformational Dynamics of the Hsp90 Molecular Chaperone Full-Length Dimer

**DOI:** 10.1371/journal.pcbi.1000323

**Published:** 2009-03-20

**Authors:** Giulia Morra, Gennady Verkhivker, Giorgio Colombo

**Affiliations:** 1 Istituto di Chimica del Riconoscimento Molecolare, Consiglio Nazionale delle Ricerche, Milano, Italy; 2 Department of Pharmaceutical Chemistry, School of Pharmacy and Center for Bioinformatics, University of Kansas, Lawrence, Kansas, United States of America; 3 Department of Pharmacology, Skaggs School of Pharmacy and Pharmaceutical Sciences, University of California San Diego, La Jolla, California, United States of America; University of California San Francisco, United States of America

## Abstract

Hsp90 is a molecular chaperone essential for protein folding and activation in normal homeostasis and stress response. ATP binding and hydrolysis facilitate Hsp90 conformational changes required for client activation. Hsp90 plays an important role in disease states, particularly in cancer, where chaperoning of the mutated and overexpressed oncoproteins is important for function. Recent studies have illuminated mechanisms related to the chaperone function. However, an atomic resolution view of Hsp90 conformational dynamics, determined by the presence of different binding partners, is critical to define communication pathways between remote residues in different domains intimately affecting the chaperone cycle. Here, we present a computational analysis of signal propagation and long-range communication pathways in Hsp90. We carried out molecular dynamics simulations of the full-length Hsp90 dimer, combined with essential dynamics, correlation analysis, and a signal propagation model. All-atom MD simulations with timescales of 70 ns have been performed for complexes with the natural substrates ATP and ADP and for the unliganded dimer. We elucidate the mechanisms of signal propagation and determine “hot spots” involved in interdomain communication pathways from the nucleotide-binding site to the C-terminal domain interface. A comprehensive computational analysis of the Hsp90 communication pathways and dynamics at atomic resolution has revealed the role of the nucleotide in effecting conformational changes, elucidating the mechanisms of signal propagation. Functionally important residues and secondary structure elements emerge as effective mediators of communication between the nucleotide-binding site and the C-terminal interface. Furthermore, we show that specific interdomain signal propagation pathways may be activated as a function of the ligand. Our results support a “conformational selection model” of the Hsp90 mechanism, whereby the protein may exist in a dynamic equilibrium between different conformational states available on the energy landscape and binding of a specific partner can bias the equilibrium toward functionally relevant complexes.

## Author Summary

Dynamic processes underlie the functions of all proteins. Hence, to understand, control, and design protein functions in the cell, we need to unravel the basic principles of protein dynamics. This is fundamental in studying the mechanisms of a specific class of proteins known as molecular chaperones, which oversee the correct conformational maturation of other proteins. In particular, molecular chaperones of the stress response machinery have become the focus of intense research, because their upregulation is responsible for the ability of tumor cells to cope with unfavorable environments. This is largely centered on the expression and function of the molecular chaperone Hsp90, which has provided an attractive target for therapeutic intervention in cancer. Experiments have shown that the chaperone functions through a nucleotide-directed conformational cycle. Here, we show that it is possible to identify the effects of nucleotide-related chemical differences on functionally relevant motions at the atomic level of resolution. The protein may fluctuate at equilibrium among different available dynamic states, and binding of a specific partner may shift the equilibrium toward the thermodynamically most stable complexes. These results provide us with important mechanistic insight for the identification of new regulatory sites and the design of possible new drugs.

## Introduction

Heat Shock Protein 90 (Hsp90) is an essential ATPase directed molecular chaperone required for folding quality control, maturation and trafficking of client proteins [1]–[4]. Hsp90 represents a fundamental hub in protein interaction networks [5],[6], with key roles in many cellular functions. Hsp90 oversees the correct maturation, activation and trafficking among specialized cellular compartments [7] of a wide range of client proteins [4],[7],[8]. The functions of clients range from signal transduction to regulatory mechanisms and immune response [3]. Client proteins typically include numerous kinases, transcription factors and other proteins that serve as nodal points in integrating cellular responses to multiple signals [3]. Given its role at the intersection of fundamental cellular pathways, it is becoming increasingly clear that Hsp90 deregulation can be associated with many pathologies ranging from cancer to protein folding disorders and neurological diseases [9],[10]. Because of this role in disease development, pharmacological suppression of Hsp90 activity has become an area of very intense research, in molecular oncology in particular. Targeted suppression of Hsp90 ATPase activity with a small molecule inhibitor, the benzoquinone ansamycin antibiotic 17-allylamino-17-demethoxygeldanamycin (17-AAG), and some of its derivatives [11],[12], has shown promising anticancer activity in preclinical models and has recently completed safety evaluation in humans [13]. Further clinical trials have also been initiated with other small molecules also used in drug combinations in various cancer types [13].

Hsp90 operates as a dimer in a complex cycle driven by ATP binding and hydrolysis and by ATP/ADP exchange. Initial structural efforts concentrated on isolated, individual domains of human [14]–[16]or yeast Hsp90 [3], [4], [17]–[21], the ER homologue Grp94 [22],[23] or the Escherichia coli homologue, HtpG [20],[24]. The crystal structures of larger constructs have also been reported [20],[25]. The first X-ray crystal structures of full-length Hsp90 from yeast bound to the ATP mimic AMPPNP revealed a homodimeric structure in which the individual protomers have a twisted parallel arrangement [26]. Each protomer, in turn, is characterized by a modular architecture with three well-defined domains: an N-terminal regulatory Domain (NTD), responsible for ATP binding, a Middle Domain (M-domain), which completes the ATPase site necessary for ATP hydrolysis and binds client proteins, and a C-terminal dimerization Domain (CTD) which is required for dimerization [26]. The same global topology is shared by the ATP-bound states of the E.coli homolog HtpG [25] and by the Endoplasmatic Reticulum (ER) paralog Grp94 [27]. Interestingly, crystal structures of the full-length constructs for Htpg or Grp94 in complex with either ADP or in the apo state showed substantially different conformations. The HtpG apo state adopted an open structure in which each of the three domains exposed hydrophobic surface area, while in the ADP-bound form these hydrophobic surfaces clustered to form a more compact state [25].

Structural and biochemical studies of the solution state of Hsp90 and its complexes using small angle X-ray scattering (SAXS) [28] have provided the first experimental evidence of a highly dynamic and stochastic nature of Hsp90, whereby the equilibrium between different conformational states of the molecular chaperone can be readily shifted to recruit a Hsp90 conformation that is suitable for efficient Cdc37 co-chaperone recognition. More recent solution structure data obtained using SAXS, single particle cryo-electron microscopy and modeling approaches showed that the apo-Hsp90 dimer (from both prokaryotic and eukaryotic sources) may be in equilibrium among different open, extended states, still preserving the constitutive dimerization provided by the CTDs, and that nucleotide binding may shift this equilibrium towards compact conformations [29]–[31]. In particular, SAXS data have revealed that the ADP-bound compact state of HtpG can be in equilibrium with an extended state, which could be significantly populated in the absence of crystal packing effects [30]. In contrast, crystal structures of AMPPNP and ADP-bound forms of the ER-paralog Grp94 showed that there is relatively little difference in conformation between the two nucleotide bound states in the crystal [27], representing extended structures. Recent studies based on mutation analysis, cross-linking and electron microscopy [32],[33] suggested that different, compact states can be accessed by Grp94 in the presence of ATP. These studies have indicated that upon binding to a specific partner, functional states of Hsp90 can be recruited using the intrinsic conformational flexibility of Hsp90.

Although the exact mechanism of coupling between ATP-binding/hydrolysis and client protein folding is still unclear, the combination of X-ray structural observations and biochemical data supports a picture in which the chaperone undergoes conformational rearrangements bringing the two NTDs in close association in the ATP-bound state, but not in the ADP-bound or apo states. This defines a conformational cycle that involves constitutive dimerization through the CTDs and transient, ATP-dependent dimerization of the NTDs in a “molecular clamp” mechanism. In terms of intrinsic protein dynamics, the mechanism of conformational coupling to the ATPase cycle involves a “tense”, structurally rigid conformational state of Hsp90 upon ATP binding, whereas a subsequent hydrolysis to ADP leads to a more “relaxed”, structurally flexible state of Hsp90 [3],[25],[26]. Finally, in the nucleotide-free form, the dimer moves to an “open” state. The crystal structures of the full-length dimer also highlight the remarkable flexibility of the ATP-lid, a segment composed of two helices and the intervening loop located immediately adjacent to the ATP binding site [26]. The lid is displaced from its position in the isolated Hsp90 NTD structure and folds over the nucleotide pocket to interact with the bound ATP yielding the ‘closed’ conformation indicating its possible importance in the progression of the chaperone cycle.

These studies are reminiscent of the results from an H/D exchange mass spectrometry investigations on the human Hsp90 in solution, which showed that the co-chaperone and inhibitor binding to the NTD can induce conformational changes at the Hsp90 domain-domain interfaces [34]. Moreover, Frey and coworkers [35] have shown that kinetic and equilibrium binding constants depend on the intrinsic conformational equilibrium of the Hsp90 obtained from different species, reflecting differential affinity and reactivity towards ATP. The kinetic analysis of the ATPase cycle has suggested that during the ATPase cycle Grp94 may be predominantly in the open state (97%). In contrast in the yeast Hsp90 the open state is only populated to 20% and a closed structure is observed in the presence of nucleotides [26]. Hence, conformational transitions during the ATPase cycle are structurally similar for different Hsp90 proteins, while the energetic balance between individual steps may be species-dependent, which is manifested in differences in the binding kinetics [35]. Overall, the solution data have suggested that the molecular mechanism of the Hsp90 chaperone cycle can be more adequately described as a stochastic process, in which ATP binding can shift the intrinsic conformational equilibrium of Hsp90 between the open apo state, the ADP-bound compact and the ATP-bound, closed protein state seen in different crystal structures.

The most recent structural studies of the apo and nucleotide-bound conformations of the E. coli, yeast, and human Hsp90 homologs have further supported the existence of a universal three-state conformational cycle for Hsp90, consisting of open-apo, ATP-closed and ADP-compact nucleotide-stabilized states, whereby the intrinsic conformational equilibrium between these states can be highly-species dependent [33]. According to these results, the evolutionary pressure may act through thermodynamic stabilization of the functionally relevant Hsp90 conformations recruited from the conformational equilibrium, to ensure the adequate response to the presence of organism-specific co-chaperones and protein clients. Importantly, ATP or ADP binding can shift the conformational equilibrium far away from the apo state for E. coli and yeast Hsp90, whereas the conformational equilibrium for human Hsp90 is largely dominated by the open form, even in the presence of the nucleotide binding. Strikingly, this study has shown that nucleotide binding provides only small stabilization energy, thereby biasing, rather than determining, the occupancy of different conformational states existing in a dynamic equilibrium.

Overall, the intrinsic conformational flexibility of Hsp90 is critical to the molecular chaperon cycle, including structural adaptation to diversity of co-chaperones and client proteins [21]. Different steps in the cycle are accompanied by binding to different co-chaperone proteins with specific functions. The Hop co-chaperone, for instance, arrests ATP hydrolysis and binds simultaneously to the Hsp70 molecular chaperone, coupling the two systems. The Hop binding to Hsp90 involves interactions at both M-domains and CTD domains [36],[37] stabilizing a conformation that is incompetent for ATP hydrolysis and N-terminal dimerization [38]. In contrast, the stress-regulated co-chaperone Aha1 substantially increases ATPase rates increasing Hsp90 chaperone activities [39]. In the case of binding to other co-chaperones, Cpr6 and Sba1, it was shown that ATP-binding and hydrolysis is required to ensure productive complex formation: interestingly, Sba1 binds to the NTD while Cpr6 binds to the CTD [40]. These observations suggest a role for the nucleotide in selecting and stabilizing different conformations of Hsp90, related to specific different functions in the chaperone cycle [33].

These crystallographic, cryo-EM, SAXS and three-dimensional single-particle reconstruction studies, applied to the isolated Hsp90 domains and full Hsp90 dimer in different species, have provided a wealth of novel insights into the molecular mechanism and function of Hsp90. However, there are still a number of important unresolved problems concerning the atomic resolution understanding of the interplay between ligand binding and the global functional motions of the molecular chaperone. We have recently performed computational studies of the Hsp90 conformational dynamics and analyzed at atomic resolution the effects of ligand binding on the energy landscape of the Hsp90 NTD by all-atom MD simulations. MD simulations of Hsp90 NTD have been carried out for the apo protein and Hsp90 complexes with its natural ligands ATP, ADP, small molecule inhibitors, and peptides [41]. These simulations have clarified the role of ATP-lid dynamics, differences in local conformational changes and global flexibility, as well as the functional interplay between protein rigidity and entropy of collective motions depending on the interacting binding partners. We have found that the energy landscape of the apo Hsp90 NTD may be populated by structurally different conformational states, featuring local conformational switching of the “ATP-lid” which is accessible on longer time scales. The results of this study have suggested a plausible molecular model for understanding the mechanisms of modulation of molecular chaperone activities by binding partners. According to this model, structural plasticity of the Hsp90 NTD can be exploited by the molecular chaperone machinery to modulate enhanced structural rigidity during ATP binding and increased protein flexibility as a consequence of the inhibitor binding. [41].

The molecular basis of signal propagation mechanisms and inter-domain communication pathways in the Hsp90 as a function of binding ligands cannot be inferred directly from crystallographic studies. As a result, computational approaches are instrumental in revealing the atomic details of inter-domain communication pathways between the nucleotide binding site and distant CTD, which may be involved in governing the chaperone equilibrium between major conformational states. In this work, we have embarked on a comprehensive computational analysis of Hsp90 dynamics and binding which provides important insights into our understanding of the Hsp90 molecular mechanisms and function at atomic resolution. We describe large-scale MD simulations to study the conformational motions and inter-domain communication pathways of the full-length yeast Hsp90 in three different complexes: with ATP, with ADP and in the apo form. In support of the experimental hypotheses, our results provide atomic models of a cross-talk between N- and C-terminal binding sites that may induce an allosteric regulation of the complex molecular chaperone machinery. These results of our study suggest that the low-resolution features of communication pathways in the Hsp90 complexes may be determined by the inherent topological architecture of the chaperone, yet specific signal communication pathways are likely to be selected and activated based on the nature of the binding partner.

## Results/Discussion

We present the results of all-atom MD simulations conducted in explicit water on the full-length yeast Hsp90 dimer, for three different complexes, representing the ATP-bound, ADP-bound and unliganded (apo) form of the protein. MD simulations have been performed using the initial crystal structure of the full-length yeast Hsp90 dimer obtained in complex with an ATP analogue and the co-chaperone p23/Sba1 [26]. It is worth noting that the difference between the starting ATP- and ADP-bound complexes entails the deletion of only one single phosphate group from a complex of more the 1200 residues. The initial apo-form is modeled by removing the ligand from the macromolecular complex followed by the minimization of the protein structure. The first objective of this work is to investigate whether these minor differences may induce global dynamical differences of the entire macromolecular machine, which can be accessed on a simulation time scale.

**Figure 1:**
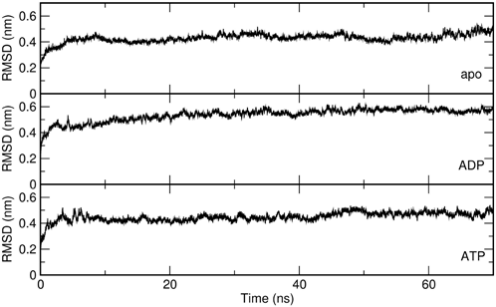
The time evolution of the RMSD values in the apo, ADP and ATP simulations. Only Cα atoms are taken into account. The model linker between NTD and M-domain, as well as the ATP lid, are not included in the calculation.

**Figure 2 pcbi-1000323-g002:**
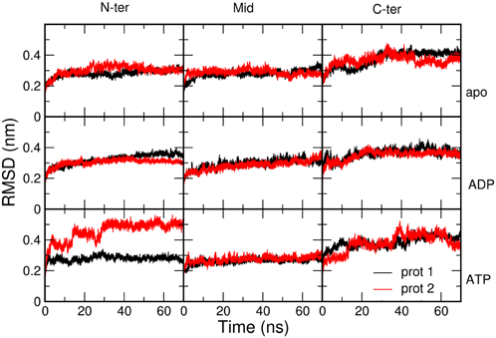
The time evolution of the RMSD values for individual domains of the dimer. Only Cα atoms are taken into account. From left to right: NTD, M-domain and CTD; from top to bottom, apo, ADP and ATP simulation.

**Figure 3:**
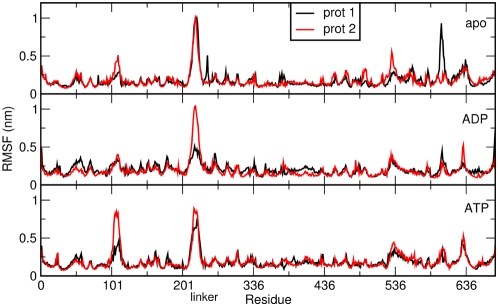
The average fluctuation per residue calculated over the 70 ns trajectory. For each simulation, the two protomers forming the dimer are plotted on the same graph, with each of them spanning residues 1–677. From top to bottom: apo, ADP and ATP simulation.

In this context, all-atom MD simulations represent a convenient method to investigate differences in global motions that are related to subtle structural and chemical changes. We have analyzed the time dependent evolution of MD trajectories for the Hsp90 dimer and separately for the individual domains by extracting fundamental structural parameters, including root mean square deviation (RMSD) for all backbone atoms (Figure 1) and the secondary structure elements (Figure 2) as well as the average residue fluctuations of the backbone residues (RMSF) (Figure 3). These data reveal substantial stability of the simulations for each of the three complexes (Figures 1–3 and Figure S1). The RMSD values of all three simulations converge to 0.4 nm–0.45 nm. However, similarity in the average global RMSD values for the three systems does not necessarily suggest that the Hsp90 dynamics evolves towards the same final structure. Rather, these values are likely to reflect structural reorganization of the molecular chaperone during the simulation course. This may be partly due to the removal of co-chaperone proteins p23/Sba1, which is known to provide additional stabilization of the Hsp90 structure. Recent experiments have shown that co-chaperone removal may induce structural rearrangements in the Hsp90 NTD [29],[33].

The most significant structural changes in simulations of the ATP-bound complex are observed in the ATP-lid region (Residues 95–120 in the NTD), paralleled by a loss of ordered helical structure in the CTD region (CTD helix 3, residues 621–636) (Figure S1). Structurally similar remodeling of the ATP-lid region has been previously detected in simulations of the isolated Hsp90 NTD [41]. The difference in the RMSD values between the NTDs from the two protomers (Figure 2) is mostly due to the local reorganization of the flexible ATP-lid and the readjustment of the position of interface residues. This observation suggests that the ATP-bound conformation of Hsp90 can populate conformations that differ from the one observed in the crystal structure, where the NTD contacts p23/Sba1 [33]. In the cases of the ADP-bound complex and apo form no significant local secondary structure transition can be observed. In particular, the secondary structure of the ATP-lid and CTD helix 3 are overall conserved during the simulation time (Figures 2 and 3 and Figure S1).

### Ligand Modulation of the Hsp90 Conformational Dynamics: Collective Protein Motions and Nucleotide-Sensitive Molecular Switching Mechanism

#### Ligand modulation of the Hsp90 collective motions

**Figure 4:**
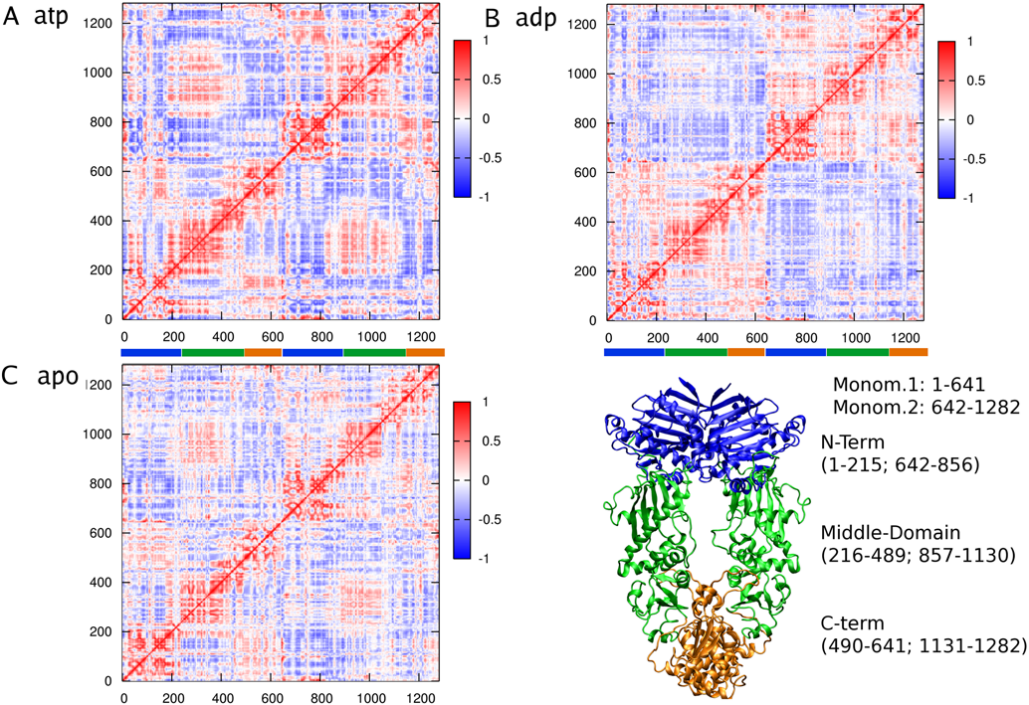
Cross-Correlation matrices calculated considering the motion of Cα atoms around the average position. A) Cross-Correlation Matrix for the Hsp90-ATP complex; B) Cross-Correlation Matrix for the Hsp90-ADP complex; C) Cross-Correlation Matrix for apo Hsp90. A correlation close to 1 (color code: yellow) corresponds to highly coordinated motion of the atom pair along the same direction, whereas a negative correlation (color code: blue) indicates motion in opposite directions. The 3D structure of the full-length Hsp90 is reported for reference, with the different domains highlighted in different colours: blue, N-terminal domain; green, M-Domain; orange, C-Domain.

We set out to quantify correlated motions within the same protomer and between two different protomers, as well as to identify protein regions that move in a concerted fashion depending on the presence of a specific ligand. In order to address these questions, we have performed analysis of the cross-correlation coefficients of pairs of Hsp90 residues from MD simulations. This approach provides a convenient framework to identify concerted, non-random fluctuations [42],[43] as a function of the ligand type. The correlation matrix describes the linear correlation between any pairs of Cα atoms as they move around their average position during dynamics (Figure 4). At a qualitative level, a positive correlation between two atoms reflects a concerted motion along the same direction, whereas a negative correlation indicates an opposite direction motion.

The correlation matrix analysis based of the ATP-bound Hsp90 complex (Figure 4A) identifies a positive correlation not only within each sub-domain, but also between different sub-domains of the same protomer, namely between the NTD and the CTD. When considering the coupling between the two protomers, not only the interacting NTD and CTD regions are positively correlated, but also the M-domains of the two protomers are found to undergo positively correlated motions (Figure 4A). The discovered correlation between the two protomers in the simulation with ATP-bound Hsp90 complex provides evidence for a globally concerted dynamics between the two protomers which, acting as rigid bodies, can bring the molecular chaperone to the “closed” state. The correlation matrix for the ATP-bound Hsp90 complex (Figure 4A) shows that the long-range positive cross-correlations can extend well beyond local elements and intra-domain regions, indicating the presence of a diffuse interaction network for the ATP-bound complex. We suggest that a functional consequence of this extensive interaction network may be to stabilize the closed “tense” state of the molecular chaperone by favoring the Hsp90 dimer complex to move as a more compact, coherent rigid body.

This picture is consistent with biochemical, X-ray crystallography [26], cryo-EM and SAXS data [25],[30] showing that ATP-binding is responsible for the close association of the two N-domains in the ATP-bound state that eventually leads to their transient dimerization in the molecular clamp mechanism. Interestingly, these results also agree with our earlier analysis of the conformational dynamics of the ATP-bound Hsp90 NTD in isolation [41], where we suggested that the effect of different binding partners on Hsp90 dynamics may propagate to the remote regions of the protein. Indeed, a unifying characteristic of the Hsp90 NTD binding-coupled dynamics was the increasing global flexibility of the domain during the hydrolysis of ATP to ADP or binding with the active inhibitors, where the conformational equilibrium of Hsp90 NTD tended to be shifted towards more relaxed, non-functional states [41].

**Figure 5:**
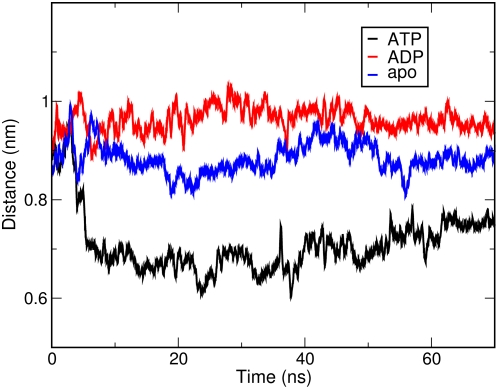
Time evolution of the N terminal interface distance. The interface distance is defined as the distance between the center of mass of the two residue groups from each protomer involved in the interface.

**Figure 6:**
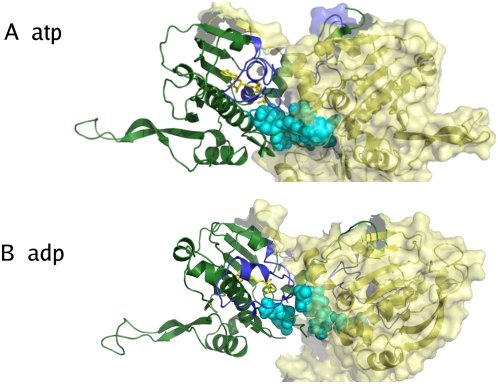
Structural representation of the N-terminal interface. The structures represent the most populated conformations obtained from the cluster analysis of the liganded dimers. The interface residues involved in hydrophobic packing are highlighted with a sphere representation (residues 22, 23, 24, 376, 378, 380). In the presence of ATP the interface becomes tighter during the simulation (A). In the ADP simulation the hydrophobic packing is disrupted (B).

In contrast, two major differences emerge from the analysis of the ADP-bound Hsp90 dynamics - the positive correlation between the two M-domains is lost and the two protomers move in an anti-correlated way (Figure 4B). However, the intra-domain positive correlation within the NTD and CTD domains is still present. The CTD helix 5 at the interface (residues 661–676) of the second protomer shows a unique behavior: it is anti-correlated with respect to the protomer it belongs to and correlated with the other one, suggesting a pivoting role in the opening mechanism (Figure 4B). The anti-correlation of the two protomers indicates collective motions towards opposite directions and consequent opening movement of the clamp (Figures 5 and 6). The correlation analysis on the apo Hsp90 dimer reveals a considerably lower degree of correlation between NTD and CTD motions, both within the same protomer and between the two different protomers (Figure 4C). Compared to the ATP and ADP cases, apo Hsp90 is characterized by less ordered (whether correlated or anti-correlated) movements, suggesting that the apo Hsp90 may exist in a dynamic conformational equilibrium between multiple states on the energy landscape. This conclusion is in accordance with the recent structural and dynamic characterization of both free and complexed structures of the bacterial Hsp90 homolog, HtpG, and the yeast Hsp90 in solution [29]–[31], where the protein is in equilibrium between the open state and a closed state.

The relative motions of the two protomers are reflected in the increased distance between the centers of mass of the hydrophobic interfaces between the two NTDs (Figures 5 and 6). Indeed, the average distance is the largest for the ADP-bound Hsp90 complex (0.96 nm), the smallest for the ATP-bound Hsp90 complex (0.71 nm) and reaches the intermediate value of 0.88 nm for the apo Hsp90 form. The salient differences in the conformational dynamics of different complexes are also reflected at the level of solvent accessible surface area (SASA) of the NTD hydrophobic interfaces: the exposed surface for the ADP-complex is 12.0 nm^2^ compared to the much lower value of 10.1 nm^2^ for the ATP-complex. The SASA value for the apo form is 10.8 nm^2^. Collectively, these data point to the evolution of the ADP-bound Hsp90 complex towards a considerably more open conformation of the chaperone as compared to the ATP-bound Hsp90 complex.

#### Nucleotide-sensitive molecular switching mechanism

**Figure 7:**
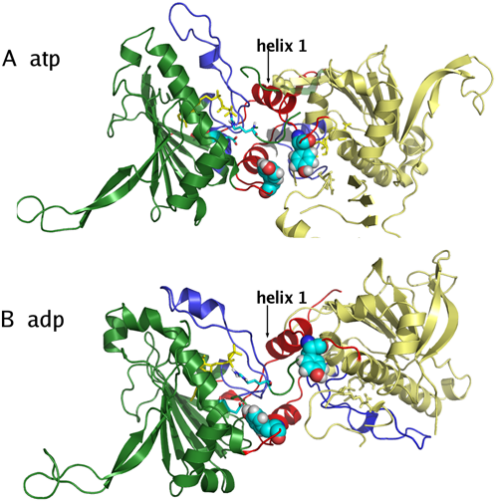
Structural detail of the interface showing the difference between the ATP and the ADP simulation. (A) In the presence of ATP the loop containing Y24 protrudes from one protomer to the other and the two tyrosines are pointing to each other. Helices 1, depicted in red in both protomers, are nearly parallel. (B) In the ADP simulation, Y24 of one protomer rotates away from the interface towards its nucleotide binding site, which results in an increased tilting of helices 1(in red) and a relative displacement of the other protomer.

The structural analysis of the three systems reveals significant differences in the local conformations and interactions of the residues from the N- and M-Domains defining the nucleotide-binding region. The residues indispensable for catalysis are E33, positioned in the NTD, and R380 located on a flexible loop in the M-Domain. In the presence of ATP, the charged side chain of R380 points towards the *γ*-phosphate of ATP in both protomers. This conformation is stabilized by a further salt-bridge interaction with E33. In one of the protomers, the nucleotide readjusts its position to stabilize a water-mediated hydrogen bonding network involving E33. This results in minor structural rearrangements in the ATP-lid and the flanking regions leading to an increased RMSD value for one of the NTDs (Figure 2). The closed conformation induced by the presence of ATP determines an increase in the number of intermolecular interactions among hydrophobic residues located on the loop connecting NTD helix 1 and helix 2, on the ATP-lid and on the middle-segment loop in the M-domain containing the catalytic residue R380. Analysis of the representative structure from the ATP-bound simulation shows that, compared to the initial crystal structure, the loop containing Y24 from one protomer extends into the groove between the NTD and the M-domain of the other protomer (Figures 6 and 7) defining tight hydrophobic packing interactions with the side chains of V23, Y24, L374, L376 and Q384 (of the other protomer), correlated to the ATP-dependent SASA decrease. The network of hydrophobic interactions is further supported by tight packing of F120 from the ATP-lid. These stabilizing interactions are further strengthened by an increase in the tilt of the domain-swapped NTD helix 1 from one protomer that ends up packing optimally with the T95, I96, and A97 sequence of the ATP-lid of the second protomer.

This analysis is corroborated by the experimental evidence that mutations of these hydrophobic residues can have a detrimental effect and result in a loss of ATPase activity [29]. It is important to underline that the new conformation attained by the Y24 loop would optimally accommodate both the T22I [26] and the T22F mutations experimentally observed to induce a 3-fold increase in Hsp90 ATPase activity (Figure S2) [29]. Both the I and F side chains, in the new conformation, would pack with the side chain of I19, N21, K27 and L129 of the same chain, further stabilizing and favoring the closed conformation by hydrophobic interactions. It is worth noting that modeling the T22F mutation in the original crystal structure (pdb entry 2CG9), determines unfavorable steric clashes and could not explain the observed increase in ATPase activity. Our data, in combination with previous experimental observations [29],[33], suggest that the hydrolysis competent solution structure may differ slightly from the X-ray structure.

**Figure 8:**
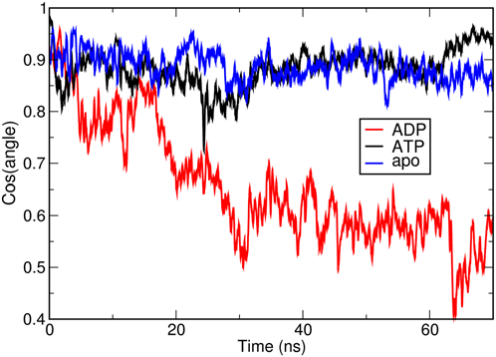
Time evolution of the tilt angle between the two N-Terminal helices 1. The figure represents the time evolution of the cosine of the dihedral angle between the axis helix 1 from one protomer and the axis of the second helix 1 from the other protomer. In the ADP simulation a clear variation of the cosine of the angle can be observed, identifying the increase of the tilt and the unwrapping of the helices described in the text.

Changing the nucleotide to ADP eliminates the interactions of the *γ*-phosphate group with E33 and R380 in the catalytic site, thereby perturbing the network of stabilizing interactions in the closed conformation. The Y24 residue rotates from its original position at the interface towards the direction of the binding site, forming a hydrogen bond with E33. This effect is reflected in the disruption of the packing interactions involving Y24 (Figure 7), and in the consequent onset of the opening motion of the dimer. The opening mechanism also determines a partial unwinding of the strap formed by the swapped N-terminal *β*-sheet and helix 1, combined with an increase of the tilt of helix 1 in the ADP-complex (Figure 8) [25]. Interestingly, recent cell biology experiments have shown that phosphorylation of Y24 appears to have a relevant impact on both the ATPase and chaperoning activity of Hsp90 (Len Neckers and Mehdi Mollapour personal communication).

Overall, these results are fully consistent with the previous computer simulations of the Hsp90 NTD dynamics [41] and experimental studies of the Hsp90 complexes with a co-chaperone and ligands [34]. Moreover, the analysis of the functionally important residues and interaction networks involved in the dynamics of the closing mechanism allows rationalizing the effects of mutations on modulation of chaperone activity that cannot be inferred from the examination of the static crystal structures.

Despite long simulation scales and the atomic level insights on the role of single important residues, the performed simulations may not have been long enough to directly observe the complete opening of the ATP-lid in the ADP-bound or apo structures and reversible transitions to the open structure of the whole dimer. However, the superposition between the representative Hsp90 NTD conformations, obtained from simulations with ADP-bound Hsp90 complex and apo Hsp90, with the corresponding crystal structures from different organisms shows that the global fold and the main secondary structure elements are highly similar (Figure S3). The main differences are localized in the ATP-lid region, where the lid may be in the open conformation, or even non-defined, in some of the crystal structures, because of its high intrinsic flexibility. Interestingly, our simulations reflect a high mobility of the ATP-lid region for the ADP-bound and apo Hsp90 forms, revealing a tendency to sample many alternative conformations.

Hence, we have found that ATP binding can bias the conformational equilibrium of the molecular chaperone towards a compact state characterized by the presence of a diffuse interaction network of residues that move in a correlated fashion. This state is compatible with the “closed” conformation, and the extensive inter-domain and inter-protomer interactions lock the complex in the “tense” state identified previously by Ali and coworkers [26]. In contrast, ADP binding determines a completely different picture in terms of correlated motions and inter-protomer interactions: domains belonging to different monomers move in a mostly anti-correlated way, corresponding to an opening motion with an increase in the distance between the two N-Domains.

### Essential Dynamics and Global Flexibility of Hsp90

To further characterize differences in the dynamic properties of the molecular chaperone, the global flexibility parameters were calculated for respective domains of the Hsp90 dimer, namely for the two NTDs, the two M-domains, and the two CTDs (Table 1). The global flexibility parameter is defined as the sum of the average fluctuations of Cα atoms during the MD trajectory. This measure of flexibility may be considered as a semi-quantitative measure of the differences in the underlying dynamics of studied Hsp90 complexes. Interestingly, the global flexibility parameters of the NTDs in the full-length Hsp90 complexes reflect a similar trend which was previously found for the isolated Hsp90 domains, namely in the presence of ATP the Hsp90 NTD domains are more rigid than in the presence of ADP or in the unbound apo Hsp90 form [41]. Hence, this analysis may capture global flexibility differences induced by the ligands in different environments: an isolated Hsp90 NTD in solution and Hsp90 NTD as an integral part of the molecular chaperone structure.

**Table 1:** Flexibility parameters for different domains of full-length Hsp90 bound to different ligands.

Flexibility [Å^2^]	N-terminal	Middle Domain	C-terminal
ATP-complex	9.0	10.1	9.3
ADP-complex	10.5	10.7	7.1
apo	9.2	11.1	11.7

Interestingly, the global flexibility parameters of the M-domains and CTD domains are also affected by the ligands. The M-domains are more rigid in the ATP-bound complex compared to the ADP-bound case, whereas for the CTDs the situation is reversed. In the presence of ATP, the CTDs show an increase in local fluctuations with respect to the simulation with ADP. The CTD interface helices (helices 4 and 5) in the ATP-complex simulation are in fact involved in hinging the rotations of the two protomers on the path to the closed conformation (see next subparagraph on ED analysis). The apo system shows an intermediate behavior at the NTD and a larger flexibility at the M-domains and CTD domains.

**Figure 9:**
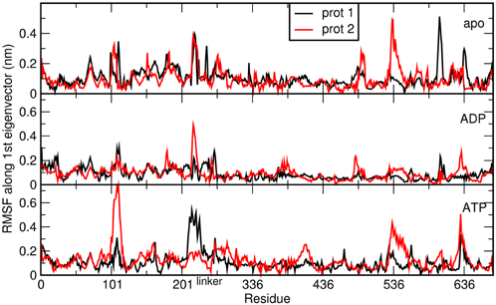
The average residue fluctuations projected on the main ED eigenvector. This quantity is calculated by projecting the trajectory over the first eigenvector according to the ED analysis. For each simulation, the two protomers (each spanning residues 1–677) forming the dimer are plotted on the same graph. From top to bottom: apo, ADP and ATP simulation.

**Figure 10:**
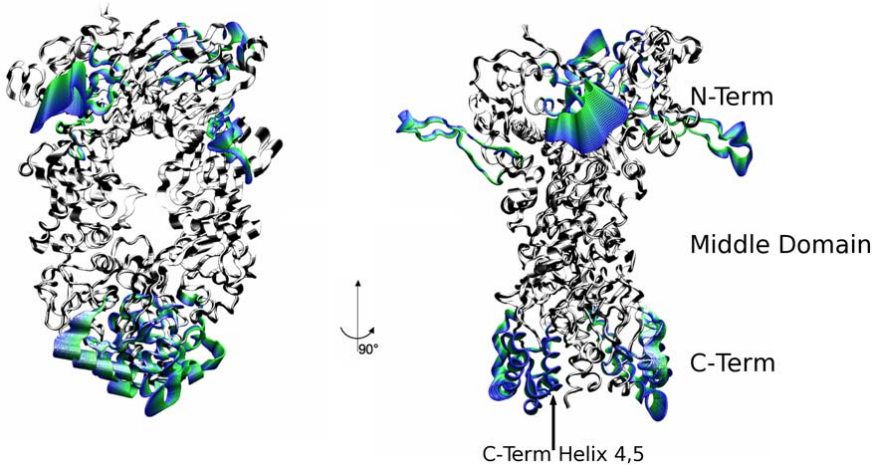
Structural analysis of the ATP-bound Hsp90. Different consecutive snapshots from the MD trajectory in the ATP simulation are projected along the main ED eigenvector and their structures superimposed. Regions undergoing relevant correlated motions are highlighted in colors; they involve the ATP lid of both protomers and helices 3 at the CTDs. The color code from green to blue reflects the time evolution of the trajectory.

**Figure 11:**
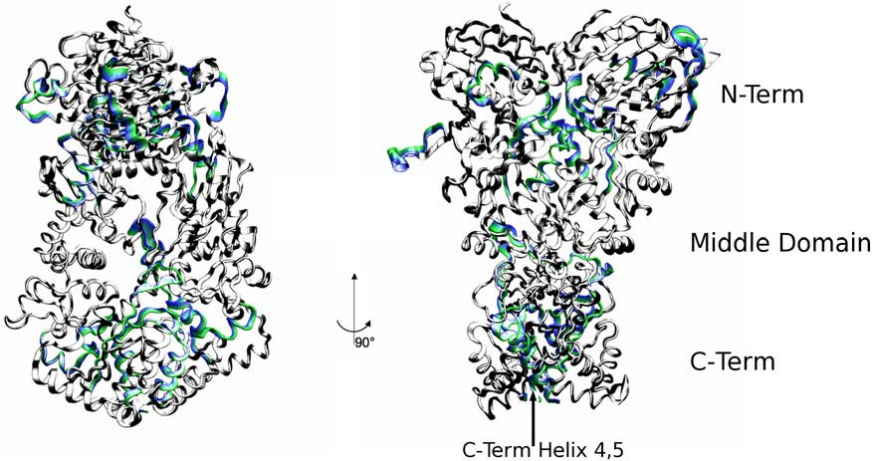
Structural analysis of the ADP-bound Hsp90. Different consecutive snapshots from the MD trajectory in the ADP simulation are projected along the main ED eigenvector and their structures superimposed. Regions undergoing relevant correlated motions are highlighted in colors. With respect to the ATP case (Figure 10), the motions are overall less pronounced and involve mainly the M-domain and the helices at the CTD interface. The color code from green to blue reflects the time evolution of the trajectory.

Essential Dynamics (ED) [44] was then used to further investigate the influence of different ligands on the dominant conformational modes of the chaperone. ED identifies functionally relevant displacements of groups of residues and emphasizes the amplitude and direction of dominant protein motions by projecting them on a subset of the principal eigenvalues and eigenvectors of the residue pair covariance matrix calculated from MD. Using this approach, we have identified the protein regions which are involved in large-scale conformational changes. In the presence of ATP, the most relevant motion involves the ATP lid at the NTD and, at the opposite end of the protein, the CTDs (Figures 9 and 10). In both protomers, the NTDs undergo a concerted rotation and compaction motion, minimizing the distance between their respective hydrophobic interfaces (Figures 10 and 11 and movies of the trajectory projections in Videos S1 and S2). The CTDs in turn rotate around the dimer interface axis. The C-terminal dimerization site is made of the two terminal helices, helix 4 and helix 5, from each protomer anchored to each other at the interface. Helix 4 is involved in the rotation of the external part of the CTD, while helix 5 acts as the hinge around which the rotation takes place. Globally, the dynamics of ATP-bound Hsp90 involves a concerted twisting and closing motion consistent with the molecular clamp model. In the presence of ADP, the projection of the MD trajectory onto the first eigenvector (Figures 9 and 11 and movies of the trajectory projections in Videos S1 and S2) shows that the main motions involve the NTD and the M-domain of Hsp90. The role of the C-terminal dimerization site is to provide the contacts necessary for the stability of the dimer either in the final open or in extended dimer conformation. At the N-terminal end, the rotation of both monomers is coupled to an increase of the inter-domain distance, as observed in the previous section. This motion propagates to the M-domains, which rigidly move, increasing their mutual distance and relative inclination. As an effect, the onset of the opening motion of the clamp is observed. Finally, for the apo protein, an opening motion from the closed conformation is observed, and is characterized by intra-domain oscillations that involve the whole chaperone, without any particular dynamic signature in terms of preferential directions of motion.

The ED analysis reflects the onset of different slow functional modes, indicating that the main features of the global motions are determined by the identity of the ligands at the binding site. Despite long simulation time scales, a direct observation of conformational transitions in the Hsp90 dimer as a function of binding partner is not yet computationally feasible. However, it is important to emphasize that functional analysis of slow modes and global motions for all the three studied Hsp90 systems is fully consistent with experimental structural and dynamical characteristics of Hsp90. For instance, our analysis shows that ATP can induce a closing motion stabilizing N-terminal dimerization, while ADP favors a transition towards a more open, relaxed state [26],[29],[30],[33]. The essential dynamics spaces (slow modes) have been shown to be exceptionally robust when calculated over trajectories of different duration, reflecting the self-similarity of the structure of the various free energy minima available to a certain system [45]. The typical amplitudes of the motions projected along the slowest modes, on the contrary, depend on the number of visited minima [45]. As a consequence, we speculate that the preferred directions of the slow modes depending on the nucleotide identity capture the fundamental properties of the biologically relevant functional modes. The possibility to extend the simulations to longer timescales could eventually allow the analysis of large-scale conformational transitions.

### Modeling Ligand-Dependent Signal Propagation Mechanisms in the Hsp90 Dimer

The results of our work have shown that the conformational dynamics of Hsp90 domains and relative stability of different conformational states can be modulated upon binding to different ligands. Importantly, we have also observed that the effect of ATP and ADP binding at the N-terminal binding site can propagate beyond the immediate binding partner and affect even remote regions of the Hsp90 dimer at a distance of more than 80 Å away from the binding interface: specifically, the fluctuations at the CTD (Figures 10 and 11) and the global motion of the M-domain depend on the nature of the ligand. The chain connectivity and organization of secondary structure elements have been identified as the main factors in determining equilibrium dynamics and regulating the mechanical tasks necessary to carry out biological functions [46]. In this context, it is of prime interest to investigate which regions and secondary structure elements of the chaperone are mostly responsible for transmitting signals coded by ATP or ADP from the N-terminal binding site to the C-terminal binding site, and whether the inter-domain communication pathways and respective signal propagation mechanisms can be modulated by the presence of the binding partner.

In order to elucidate the mechanisms of signal propagation from the nucleotide binding site, we have extended and adapted a recent approach proposed by Bahar and coworkers [47],[48] to the analysis of all-atom MD simulation trajectories. The analysis of signal propagation, which was developed based on elastic network models [47],[48], describes signal transduction events in proteins as directly related to the fluctuation dynamics of atoms, defining the communication propensity of a pair of residues as a function of the fluctuations of inter-residue distance. In the present study, we investigate the presence of long-range communication propensities between NTD residues close to the nucleotide binding site and remote regions of the protein, such as the CTD interface.

**Figure 12:**
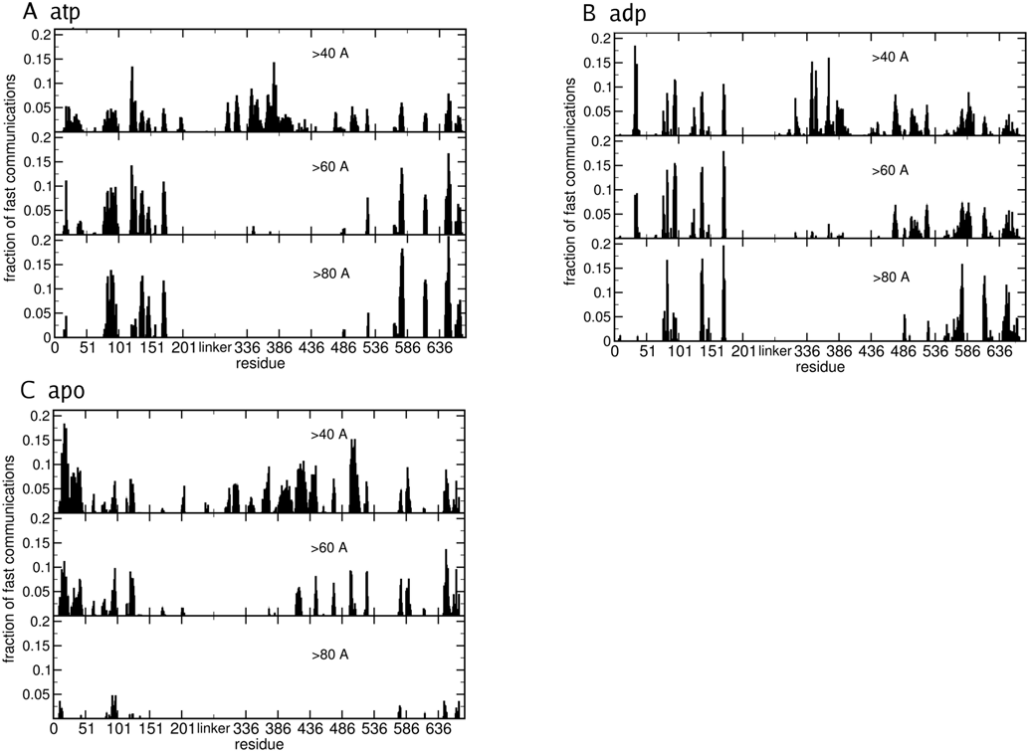
The histogram analysis of the communication efficiency. (A) The histogram for the Hsp90-ATP complex; (B) The histogram for the Hsp90-ADP complex; (C) The histogram for apo Hsp90. Each bin refers to a residue and shows the fraction of residues of the whole protein that are highly prone to communicate with it, i.e. such that their pair CP value is below 0.025. In each histogram only communications at distances greater than a given threshold are considered, namely over 40, over 60 and over 80 Angstroms.

**Figure 13:**
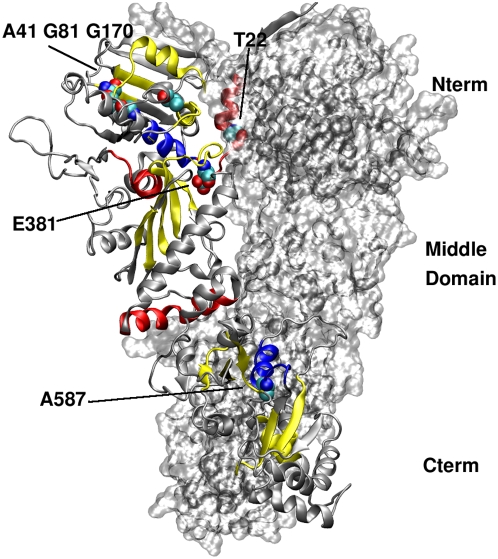
Structural analysis of the communicating residues in the Hsp90 dimer. Residues active in long range signalling (i.e. active in communication with other residues that are more than 40 Angstroms apart) are coloured according to the following scheme: yellow: Residues active both in ATP and ADP system. Red: Residues active in the ATP system. Blue: Residues active in the ADP system. Mutation sites leading to different phenotypes [49] are highlighted with spheres and labelled.

The communication propensity (CP) is calculated for any pair of residues during the trajectory. It is worth noting that CP describes a communication time, therefore low CP values are related to efficiently communicating residues. The average CP value for consecutive amino acids along the sequence, calculated considering for each residue *i* the neighbors comprised between *i−4* and *i+4*, is 0.025. The average CP value for residues distant more than 40 Å is 0.12. In the ADP and ATP simulations, around 1 percent of residue pairs have CP<0.025 even if they are at distances larger than 40 Å, while the percentage decreases to 0.5 percent in the unbound complex (Figure S4). Therefore, in the presence of ligands, a number of very distant residues may have a high communication propensity despite their physical separation and we set CP = 0.025 as a convenient threshold for discriminating fast communications at long distance. In order to investigate this aspect more thoroughly, for each complex (ATP-bound, ADP-bound and apo) a set of histograms was created, in order to scan communication efficiencies at increasing distances (Figure 12). Each bin refers to a residue and gives the fraction of residues that have high communication efficiency with it (CP<0.025) at distances larger than an increasing cutoff of 40 Å, 60Å and 80 Å respectively. Residues corresponding to histogram peaks define regions that are specifically involved in efficient long-range communications. The histogram at 40 Å indicates that residues active in long range signaling belong to NTD, at the M-domain and CTD of the dimer. According to the experimental data, a number of experimentally studied mutations, known to determine temperature sensitive phenotypes [49], and therefore perturbing functionally relevant regions, involve residues which in the present analysis are related to peaks of efficient signal communication (Figures 12 and 13). For instance, the T101I mutant has normal ATP affinity but reduced ATPase activity, whereas the mutant T22I displays enhanced ATPase activity and AMP-PNP-dependent NTD dimerization [50]. Also mutation of several M-domain residues (Arg-376, Gln-380 in yeast Hsp90) has been shown to affect ATPase activity [51] whereas mutants A587T and T101I of yeast Hsp90 render cells much more sensitive to Hsp90 inhibitor drugs [52]. Sites T22, A41, G81, G170, E381 and A587 correspond to peaks in both the ATP and ADP bound cases (Figures 12 and 13). Two of them, namely T22 and G81, as well as residues adjacent to T101, correspond to N-Domain long range communication sites, whose signaling properties persist at very long distances, as discussed in the following. Upon increase of the residue-residue distance in the CP scanning histograms, some peaks become progressively smaller or disappear, since the fraction of effectively coordinated residues decreases at longer physical distances. On the other hand, since the total number of possible pairs also decreases with increasing distance, for some residues the fraction of efficient communications may grow at longer distances, and those residues we define to be strongly active in long range signaling.

**Figure 14:**
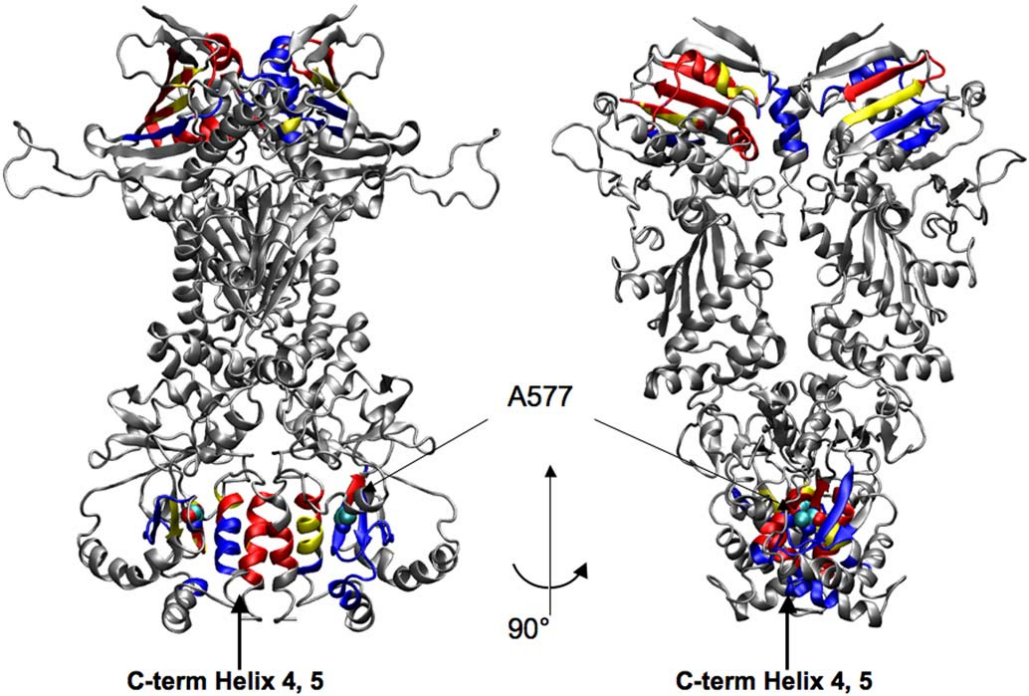
Representation on the 3D structure of residues communicating with high efficiency. Two views of Hsp90 dimer, where residues active in very long range signalling (i.e. active in communication with other residues that are more than 80 Angstroms apart) are coloured according to the following scheme: Yellow: Residues active both in ATP and ADP system. Red: Residues active in the ATP system. Blue: Residues active in the ADP system. The mutation site A577 leading to loss of the ATPase activity is highlighted with spheres and labelled.

The important finding from our analysis is that in the absence of any ligands, all peaks decrease at increasing distances, while in the presence of both ATP and ADP a number of peaks grow when distance increases. The most efficient communications at very long distance (over 80 Å) involve a subset of specific residues in all complexes, while for the apo form of Hsp90 only a small fraction of these residues is active. Moreover, we observe that in the presence of ATP the long-range communication from the binding site is mainly directed to specific residues at the CTD interface, while ADP activates communications between the NTD binding region and the CTD region surrounding the interface (Figure 14).

In simulations with the ATP-bound Hsp90 complex, the NTD residues 81–95 and 121–140 (Hsp90 residues numbering as in the pdb entry 2CG9) show a high long-range signaling propensity with segments 574–580 and with the two C-terminal interface helices, made of residues 645–654 (helix 4) and 661–671 (helix 5) respectively. In simulations with the ADP-bound Hsp90 complex, the NTD residues 75–83 and 91–96 communicate with the C-terminal regions comprising residues 566–578, 611–625 and again the helical segment 645–654 (helix 4) (Figure 14). In agreement with our previous results, this analysis suggests that ATP and ADP may have a different effect on the Hsp90 conformational dynamics, which is manifested in the activation of differential signal propagation pathways. However, both ATP and ADP may activate the inter-domain communication between the NTD and the β-sheets adjacent to the C-terminal interface. The A577C mutation in this β-sheet (Figure 14) coupled with successive S-nitrosilation, was experimentally shown to affect the ATPase activity of the chaperone (Marco Retzlaff and Johannes Buchner, personal communication). It is important to emphasize that the role of specific point mutations Y24F and A577C, affecting the inter-domain signal propagation, was independently validated by different experimental groups (Len Neckers, Marco Retzlaff and Johannes Buchner and personal communication).

Inhibition of the ATPase activity of Hsp90 by either mutation or small molecule inhibitors results in the degradation of client proteins in vivo, demonstrating the central importance of ATPase activity to the function of the chaperone [53]. The evidence of an efficient molecular communication between NTD residues near the binding site and CTD regions at the interface is of special importance given the rapidly growing interest in developing novel and specific Hsp90 inhibitors inhibition targeting allosteric Hsp90 regions, including CTD. It has been shown that the binding of coumarin-type antibiotics, such as novobiocin, at the carboxyl terminus antagonizes geldanamycin binding at the NTD [54], blocks the binding of immunophilin co-chaperones *in vitro* [55] and markedly reduces the cellular levels of several oncogenic proteins kinases *in vivo* [56]. Also, inhibitor binding to the NTD site affects the binding of co-chaperones at the C-terminal site whereas the presence of co-chaperones there impacts negatively on the N-terminal binding of nucleotides or inhibitors [54]–[56]. The protein segment found to be involved in the binding of C-terminal inhibitors has sequence YETALLSSGFSLED in human Hsp90 [54],[56] and corresponds to residues 647 to 660 in yeast Hsp90. This segment is located at the interface helices (helix 4 and subsequent loop connecting to helix 5) that we define to be active in communicating with the N-Domain binding site in the ATP simulation (Figure 14). Hence, the inter-domain communication from the NTD to the C-terminal interface is visible only in the presence of ATP (Figure 14). Our data therefore support, at atomic-resolution, the picture of a cross-talk between N- and C-terminal binding sites inducing allosteric regulation, as hypothesized by experimental observations.

The method of CP analysis is based on the hypothesis that signal propagation in complex molecular systems can inflicted by correlated fluctuations determined by the intrinsic network topology. The ordered fluctuations are coupled to the global dynamics sampled under equilibrium conditions, thus controlling allosteric effects. In the original formulation, based on a coarse-grained representation of the protein, the Gaussian Network Model (GNM) [47],[48], provides an elegant, yet largely qualitative characterization of signal transduction pathways. Our study is an extension of this approach to the analysis of long MD simulations and allows to include all-atom representations and the detailed energetics of the system, that more adequately reflect the effect of subtle, yet important chemical modifications. These results suggest that the coarse-grained features of communication patterns may be dependent on the inherent topological architecture and packing of the protein [45]–[48], [57]–[59], but also that specific pathways can be selected and activated based on the binding partner. We speculate that structural architecture of the molecular chaperone and the intrinsic dynamic equilibrium between major conformational states can define the topology and energetics of signal communication pathways, which may be modulated by the binding partner.

### Gaussian Network Model Analysis

The correlation analysis based on all-atom MD simulations was compared with the results of the elastic network (EN) and GNM approaches. These coarse-grained models are widely used for elucidating the collective dynamics of proteins and exploring their relevance to biological function [60],[61]. The basic ingredient in the GNM models is the topology of the inter-residue contacts in the native structure, which appears to be the major determinant of equilibrium dynamics [60],[61]. We have found that the correlation matrix obtained from the GNM analysis applied to the full-length dimer of Hsp90, in the absence of ligands, is consistent with that obtained from MD simulations. Namely, it reveals a positive correlation between the NTD and CTD within each protomer, which may be considered as an intrinsic feature of the three-dimensional structure of the system. Within the GNM model, the inter-protomer correlation between the two M-domains is not seen, which is in agreement with the results obtained from the all MD simulations of the apo Hsp90. Importantly, we have confirmed that signal propagation pathways emerging from the GNM model [47] are similar to the ones inferred from the all-atom description of the system (Figures S5 and S6). In this context, it should be noted that GNM methods are limited by the lack of information on residue specificities (effects of mutations), atomic details (chemical modifications) or side chain motions. ATP and ADP are very similar molecules and the simplification of their representation in terms of interaction centers, consistent with the GNM approach used here, would not allow to explicitly distinguish between different complexes, starting from the same chaperone structure. In this context, all-atom MD simulations allow to differentiate the effects of inter-domain correlations resulting from subtle chemical differences between ATP and ADP.

For a further comparative analysis of all-atom and GNM representations of the system, we have selected the representative Hsp90 conformations from the most populated structural clusters obtained from ATP-bound and ADP-bound simulations. These protein conformations are then used in the GNM model to elucidate the main chain collective motions. Interestingly, the GNM analysis of the representative ATP-bound structure produces slow modes reflecting a closing motion involving the NTD and CTD. In contrast, a coarse-grained analysis of the ADP-bound representative conformation encodes for an opening motion of the two NTDs (data not shown). Hence, these complementary models have recapitulated major features of the Hsp90 conformational cycle consistent with the “molecular clamp” mechanism. A combination of all-atom MD and GNM approaches may be a promising approach for exploring large conformational changes in complex molecular systems [62].

### Conformational Selection Model of the Hsp90 Dynamics

The nucleotide-dependent collective motions observed in our study recapitulate the essential atomic-level determinants of the molecular-clamp mechanism: ATP-binding brings the molecular chaperone into a closed, “tense” state characterized by an extended network of coherently moving residues, a compaction of the hydrophobic inter-protomer interfaces at the NTD and M-domain and determines a principal collective motional mode compatible with the closure of the clamp. ATP hydrolysis or ADP binding induces a high degree of anti-correlation in the inter-protomer motions, and the principal collective mode leads to a transition towards the “open” state of the molecular clamp. The two NTDs move apart and the NTD and M-Domain hydrophobic interfaces begin to be disrupted, the number of contacts decreases and their interaction is looser. This picture is consistent with a conformational change in the direction of the extended (or completely open) structure of the dimer. The collective motions of the apo Hsp90 structure do not reveal any specific signature, suggesting that its dynamics may be readily biased towards different regions of the conformational space upon ligand binding.

Within the limitations of all-atom MD simulations, the results of our analysis suggest possible links between local interactions and short timescale fluctuations with slower biologically relevant functional motions. The results of our simulations refer to timescales of tens of nanoseconds and report on the microscopic behavior of the chaperone complexed to different ligands, while functional motions may take place on timescales that are orders of magnitude higher. It is worth noting at this point that in order to reach the states competent for client binding, folding or release, Hsp90 structures have to undergo extensive reorganization in an efficient fashion. One possible way to achieve this goal would be to sample pre-organized states and motions that are dictated by the local organization of interactions and by the local correlations/fluctuations described above and modulated by different nucleotides. This mechanism would define a hierarchy of possible dynamic substates that the chaperone can search in a more efficient way than by random sampling of all possible conformations. The concept of hierarchy of substates has already been investigated previously [63]–[65], together with the idea that preferred relatively small, local fluctuations in enzyme systems lead to states resembling the catalytically active conformations. In this framework, Henzler-Wildman et al. in a fundamental study on the enzyme Adenylate Kinase (Adk) [66], correlated smaller amplitude, faster motions (ps to ns) at the atomic scale with the large amplitude, slow motions on the micro- to millisecond scales related to enzymatic reactivity. The results of our simulations suggest that global rearrangements of Hsp90, occurring on longer timescales, can be facilitated by nucleotide-dependent microscopic motions and correlated fluctuations occurring on the shorter timescales that are now accessible to all-atom MD simulations.

The “conformational selection model” [67] appears to be the most suitable model to explain the functional dynamics of the full-length Hsp90 dimer. The protein fluctuates at equilibrium among different dynamic states available on the energy landscape, and binding of a specific partner may shift the equilibrium towards thermodynamically most stable complexes. Local microscopic fluctuations accessible in the MD timescale facilitate the large-scale, slower global motions that regulate the chaperone cycle [68]. Different parts of the N-Domain are involved in sensing the presence of a certain specific ligand and switching to the specific phase of the chaperone cycles. The formation of this complex is sensed by the ATP-lid, and the interfaces of the N- and C-Domains, that couple nucleotide binding and hydrolysis to global dynamics and hydrophobic surface remodeling. These considerations are consistent with the energy landscape theory, whose application is extended here to macromolecular machines, for which coupling between dynamics and binding is often accompanied by conformational transitions associated with the biological functions of proteins and as such are intimately connected to the underlying energy landscape. These results also recapitulate the notion that binding of different ligands modulates the degree of local energetic frustration of the Hsp90 interface regions that are important for function [69], i.e. the hydrophobic inter-protomer interface between the N-Domains and M-Domain, favoring or disfavoring the formation of stable inter-protomer interactions. The structural plasticity observed in our study may favor adaptation of the chaperone to bind with sufficient affinity the wide range of protein clients with different sequences and conformations. These findings are in accordance with the recent results advocating a potential universal role of conformational selection mechanisms in a variety of biological systems [67], [70]–[72].

### Conclusions

In this study, we have presented a comprehensive molecular analysis of signal propagation mechanisms and long-range communication pathways in the molecular chaperone Hsp90. The analysis is carried out based on large-scale all-atom MD simulations of Hsp90 molecular chaperone full-length dimer in explicit water. We have supplemented these simulations with a battery of computational modeling and analysis tools, including essential dynamics, correlated motions analysis and signal propagation modeling. The results of our simulations shed light on functionally relevant motions of the full-length Hsp90, recapitulating fundamental features of the chaperone conformational cycle consistent with the “molecular clamp” mechanism. We have elucidated the mechanisms of signal propagation and discovered functionally important residues and secondary structure elements that emerge as hot spot mediators of communication between the nucleotide-binding site and the C-terminal interface.

Furthermore, we have fond that inter-domain signal propagation pathways may be activated as a function of the binding partner. In particular, ATP binding may switch on a rapid communication between the ATP-lid, the nucleotide binding site, the catalytic loop of the M-Domain and the two helices defining the interface between two CTDs from the two different monomers. In contrast, the presence of ADP defines a different signal transduction path involving the main helix of the NTD, and the external helices of the CTD.

We have provided a comprehensive computational analysis of the Hsp90 communication pathways and conformational dynamics at atomic resolution, revealing the role of the nucleotide in effecting conformational changes. The results of this study support a “conformational selection model” of the Hsp90 binding mechanism, whereby the protein may exist in a dynamic equilibrium between different conformational states available on the energy landscape, and binding of a specific partner can bias the equilibrium towards functionally relevant complexes.

## Methods

### Molecular Dynamics of Hsp90 Complexes

All-atom MD simulations in explicit water have been independently carried out on a long simulation time scale of 70 ns for Yeast Hsp90 dimer complexed with the natural substrates ATP and ADP and for the unbound dimer (apo form). The crystal structure (pdb entry 2CG9) [26] containing Yeast Hsp90 dimer bound to ATP and complexed with co-chaperone p23/Sba1 has been used as starting conformation, upon removal of the co-chaperone. The crystal structure employed as a starting point for the simulations was obtained by the deletion of the long disordered loop connecting the NTD and the M-domain and by introducing the Ala107Asn point mutation, that was used to stabilize ATP-binding and the closed structure in order to obtain more favorable crystallization conditions [26]. These two factors were shown not to have an impact on the general functional properties of the chaperone [26]. However, the charged loop connecting the NTD and the M-domain, which is not present in the crystal structure, is replaced by a model linker made of 10 Gly residues and modeled by the ModLoop server. Also disordered loops in the CTD are modeled with ModLoop, but conserving the original sequence [73]. The p23/Sba1 co-chaperone atoms were removed from the starting structures used in the simulations. The crystal structure also included the p23/Sba1 co-chaperone protein [26], which may also contribute to the stabilization of the closed Hsp90 structure. In simulations, the p23/Sba1 co-chaperone atoms were completely removed from the starting structures.

The crystal structure ( pdb entry 2CG9) does not contain Mg^2+^ ions, most likely due to the resolution at which the crystals were obtained, which is too low to exactly localize the ion. Mg^2+^ is required for activity in many ATPases and is also present in the 1Y4S structure of the HtpG, where it coordinates ADP. Mg^2+^ ions were not included in our simulations. This choice was due to the absence of the coordinates for the ion in the X-ray data from the pdb due to low resolution, and to uncertainties related in placing the ion in the active site either by hand or by using automated docking programs. Furthermore, it was shown previously that the introduction of a small number of ions can lead to very severe sampling and equilibration problems suggesting that in practical calculations convergence can best be achieved by incorporating either no counter ions or by simulating at high ionic strength to ensure sufficient sampling of the ion distribution [74]. Finally, in runs on the isolated NTD domains used for the study reported in [41] we noticed no substantial differences on the global dynamic properties, at least in the time scales simulated, in the case of presence or absence of Mg^2+^.

The apo and ADP simulations have been carried out respectively by removing the ligand and by replacing ATP with ADP, as in [41]. The tetrahedral solvation box contains around 57000 particles. All simulations and the analysis of the trajectories have been performed using the GROMACS software package [75] using the GROMOS96 force field [76] and the SPC water model [77].

Each Hsp90 dimer system simulated in this study was first energy relaxed with 2000 steps of steepest descent energy minimization followed by another 2000 steps of conjugate gradient energy minimization. The energy minimization was used to remove possible bad contacts from the initial structures. For each of the simulations, the system was equilibrated by 50 ps of MD runs with position restraints on the protein and ligand to allow relaxation of the solvent molecules. These first equilibration runs were followed by other 50 ps runs without position restraints on the solute. The first 5 ns of each trajectory were not used in the subsequent analysis in order to minimize convergence artifacts. Equilibration of the trajectories was checked by monitoring the equilibration of quantities such as the RMSD with respect to the initial structure, internal protein energy, fluctuations calculated on different time-intervals. The electrostatic term was described by using the particle mesh Ewald algorithm. The LINCS [78] algorithm was used to constrain all bond lengths. For the water molecules the SETTLE algorithm [79] was used. A dielectric permittivity, ε = 1, and a time step of 2 fs were used. All atoms were given an initial velocity obtained from a Maxwellian distribution at the desired initial temperature of 300 K. The density of the system was adjusted performing the first equilibration runs at NPT condition by weak coupling to a bath of constant pressure (P_0_ = 1 bar, coupling time τ*_P_* = 0.5 ps) [80]. In all simulations the temperature was maintained close to the intended values by weak coupling to an external temperature bath [80] with a coupling constant of 0.1 ps. The proteins and the rest of the system were coupled separately to the temperature bath. The structural cluster analysis was carried out using the method described by Daura and coworkers with a cutoff of 0.25 nm [81].

### Correlation and Essential Dynamics Analysis

Principal Component Analysis or Essential Dynamics (ED) reduces the dimensionality of the covariance matrix by diagonalization [44]. This method describes global protein motions that are represented by the matrix eigenvectors and eigenvalues. Principal eigenvectors are in general associated with large-scale movements, also termed slow modes, which are responsible for protein functions.

The covariance matrix for each of the simulated Hsp90 dimer systems was built by averaging motions of Cα atoms deviating from the mean structure, with the latter calculated over the trajectory excluding the first 5 ns needed for equilibration. Ligands are not included in the calculation. Translational and rotational degrees of freedom are eliminated and the average atomic coordinates, *x_i,ave_*, i = 1,…,3N, are calculated along the MD trajectory (20). The essential directions of correlated motions during dynamics are then calculated by diagonalizing the 3N×3N covariance matrix *C_ij_*.
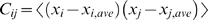
The MD trajectory can be projected onto the main essential direction, corresponding to the largest eigenvector, in order to visualize the extreme structures and the major fluctuations of the correlated motions. The correlation matrix *Corr_ij_* is a N×N array, whose i-j entry summarizes the correlation between the motion of atom i and of atom j, is obtained from the reduction and normalization of the covariance matrix.
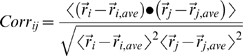



### Signal Propagation Analysis

We have extended and adapted a recent approach proposed by Bahar and coworkers to the analysis of all-atom MD simulation trajectories. The analysis of signal propagation, which was developed based on elastic network models [47], defines signal transduction events in proteins as directly related to the fluctuation dynamics of atoms, defining the communication propensities (CP) of a pair of residues as a function of the fluctuations of interresidue distances. Residues whose C*α*-C*α* distance fluctuates with a relatively small intensity during the trajectory are supposed to communicate more efficiently than residues whose distance fluctuations are large. In the former case, a perturbation at the one site, affecting the C*α* position, is likely to be visible (reflected) at the second site, while in the latter case the communication is less efficient, due to the intrinsic amplitude of the distance fluctuations. The Communication Propensity (CP) of any two residues is defined as the mean-square fluctuation of the interresidue distance defining 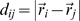 as distance between the C*α* atoms of residue *i* and residue *j*, respectively:
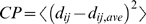
By projecting these quantities on the 3D structures of the protein bound to different ligands, it will be possible to identify possible differences in the inter-domain and inter-protomer long-range redistributions of interactions.

### Coarse-Grained Modeling

We employed the implementation of the GNM approach developed by Micheletti and coworkers [82] to identify collective conformational changes and large-scale movements for the apo-protein, and compare the main slow modes to those identified by the all-atom MD simuation model. This model implements a Cα-based amino acid representation of the protein. The energy function used to simulate the thermal equilibrium fluctuations of the protein around the reference conformation is obtained by introducing the following penalties for displacing two Cα's, *i* and *j* from their reference positions, r*_i_* ^0^ and r*_j_* ^0^ to generic ones r*_i_* and r*_j_*.
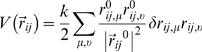
where r^0^ *_ij_* is the distance vector, 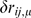 is the distance vector change, ν and μ and run over the three Cartesian components and *k* is a parameter controlling the strength of the quadratic coupling. The form of the energy function in equation 2 is common in most ENM or GNM approaches.

Exploring the dynamics of the protein complexes in this simplified theoretical framework allows calculating the degree of correlation of the displacement from the equilibrium position of pairs of residues. Communication Propensities in this framework are calculated according to [47].

## Supporting Information

Figure S1. The time evolution of the secondary structure according to the DSSP definitions.
(0.56 MB TIF)

Figure S2 Structural packing of the T22F mutant. The mutant is modelled in the most representative conformation from the ATP-complex simulation. F22 is represented as van der Waals spheres together with the side chains of contacting residues. The optimal hydrophobic packing is evidenced.
(2.25 MB TIF)

Figure S3 The superposition between a representative NTD conformation from the ADP (a) and apo (b) simulations, with the corresponding crystal structure from different organisms. For the crystal structure, only the coordinates of lid region and of Tyr24, when present, are explicitly represented. (a) ADP 1. The superposition between a representative conformation from the simulation (green) and crystal structure of N-terminal domain bound to ADP (PDB 1AMW.pdb, violet). 2. The superposition between a representative conformation from the simulation (green) and crystal structure of HtpG (PDB 2IOP, red). 3. The superposition between a representative conformation from the simulation (green) and crystal structure of Grp94 (PDB 1TC6, orange). (b) APO 1. The superposition between a representative conformation from the simulation (green) and crystal structure of apo HtpG (PDB 2IOQ, red). 2. The superposition between a representative conformation from the simulation (green) and crystal structure of apo Grp94 N-terminal domain (PDB 1YT2, orange).
(5.78 MB TIF)

Figure S4 The Histogram showing the number of fast communication events (CP<0.025) for each given distance of the communicating residues. In the ATP and ADP complexes the inter-domain communications persist at extremely long range (over 80Å); while such a long distance tail is not present in the apo system.
(2.25 MB TIF)

Figure S5 The cross-correlation matrix calculated of the Hsp90 dimer using the GNM approach. A correlation close to 1 (color code: yellow) corresponds to a highly coordinated motion of the atom pair along the same direction, whereas a negative correlation (color code: blue) indicates motion in opposite directions.
(2.25 MB TIF)

Figure S6 The analysis of inter-domain communication pathways in the Hsp90 dimer using the GNM approach. A Set of histograms showing the communication efficiency of all residues, evaluated at increasing distances. Each bin refers to a residue and shows the fraction of residues that are highly prone to communicate with it, i.e. such that their pair CP value is below 2.5. In each histogram only communications at distances greater than a given threshold are considered, namely over 40, over 60 and over 80 Angstroms.
(2.25 MB TIF)

Video S1 Ligand modulation of global essential movements in the ATP-complex: closing motion.
(0.28 MB MOV)

Video S2 Ligand modulation of global essential movements in the ADP-complex: opening motion.
(0.28 MB MOV)
