# Supplementary figures and images for: Modeling Signal Propagation Mechanisms and Ligand-Based Conformational Dynamics of the Hsp90 Molecular Chaperone Full-Length Dimer
Source: PLoS Comput Biol. 2009 Mar 20;5(3):e1000323. doi: 10.1371/journal.pcbi.1000323 (PMC2649446; doi:10.1371/journal.pcbi.1000323)

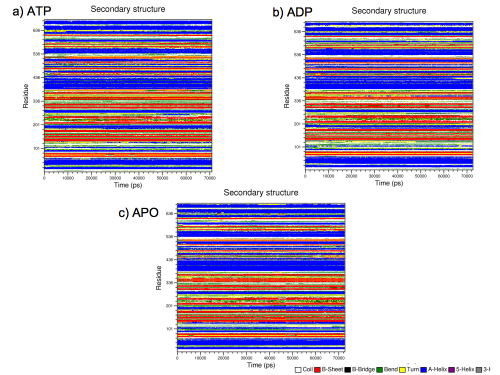

Supplement: Figure S1. — The time evolution of the secondary structure according to the DSSP definitions. (0.56 MB TIF) [file pcbi.1000323.s001.tif]

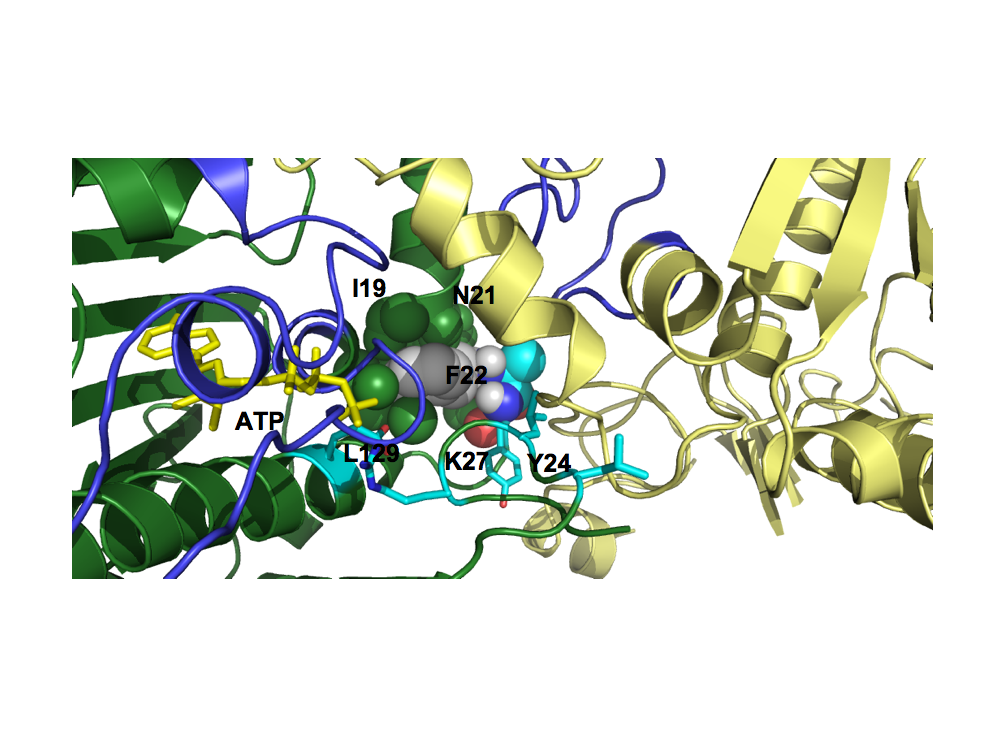

Supplement: Figure S2 — Structural packing of the T22F mutant. The mutant is modelled in the most representative conformation from the ATP-complex simulation. F22 is represented as van der Waals spheres together with the side chains of contacting residues. The optimal hydrophobic packing is evidenced. (2.25 MB TIF) [file pcbi.1000323.s002.tif]

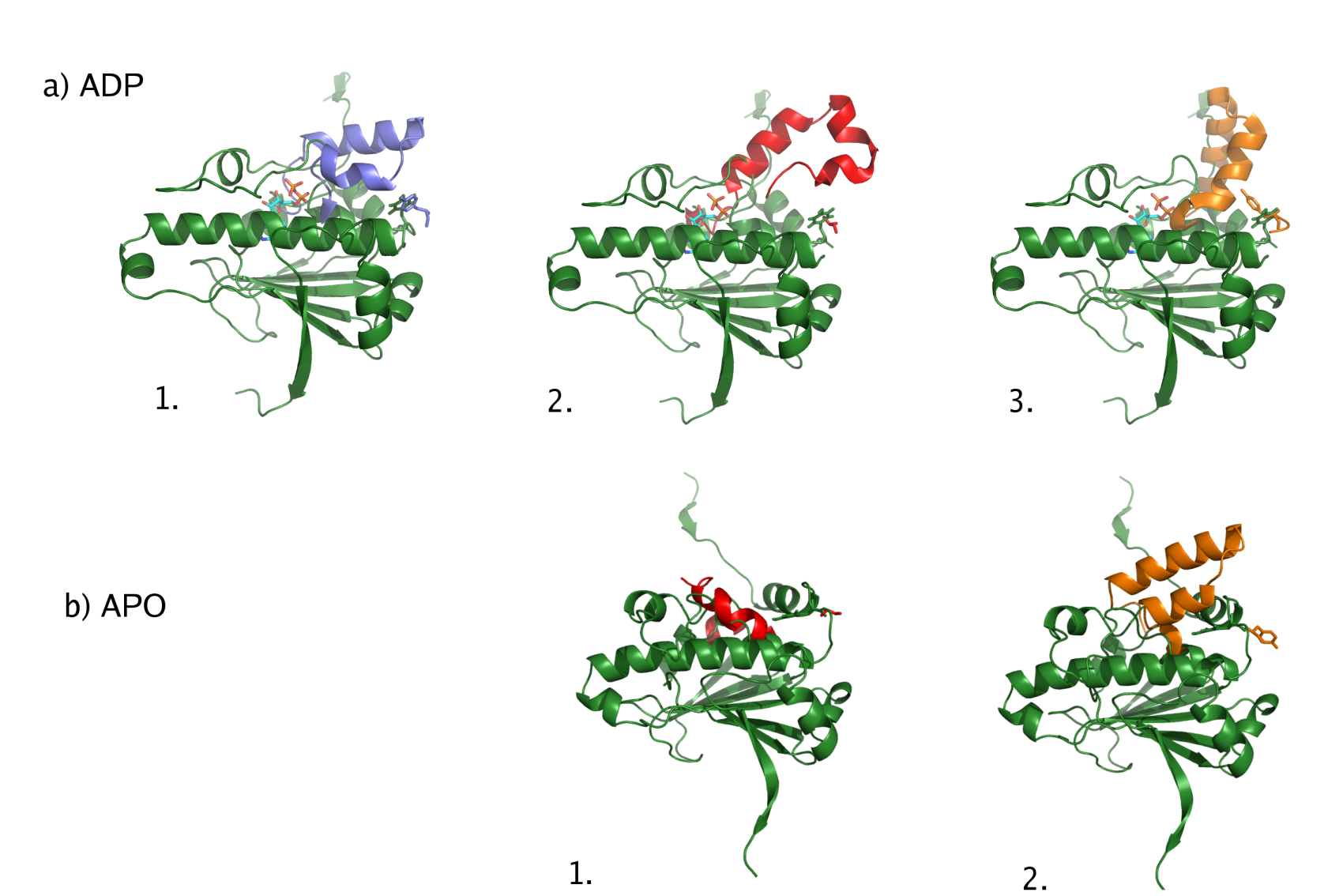

Supplement: Figure S3 — The superposition between a representative NTD conformation from the ADP (a) and apo (b) simulations, with the corresponding crystal structure from different organisms. For the crystal structure, only the coordinates of lid region and of Tyr24, when present, are explicitly represented. (a) ADP 1. The superposition between a representative conformation from the simulation (green) and crystal structure of N-terminal domain bound to ADP (PDB 1AMW.pdb, violet). 2. The superposition between a representative conformation from the simulation (green) and crystal structure of HtpG (PDB 2IOP, red). 3. The superposition between a representative conformation from the simulation (green) and crystal structure of Grp94 (PDB 1TC6, orange). (b) APO 1. The superposition between a representative conformation from the simulation (green) and crystal structure of apo HtpG (PDB 2IOQ, red). 2. The superposition between a representative conformation from the simulation (green) and crystal structure of apo Grp94 N-terminal domain (PDB 1YT2, orange). (5.78 MB TIF) [file pcbi.1000323.s003.tif]

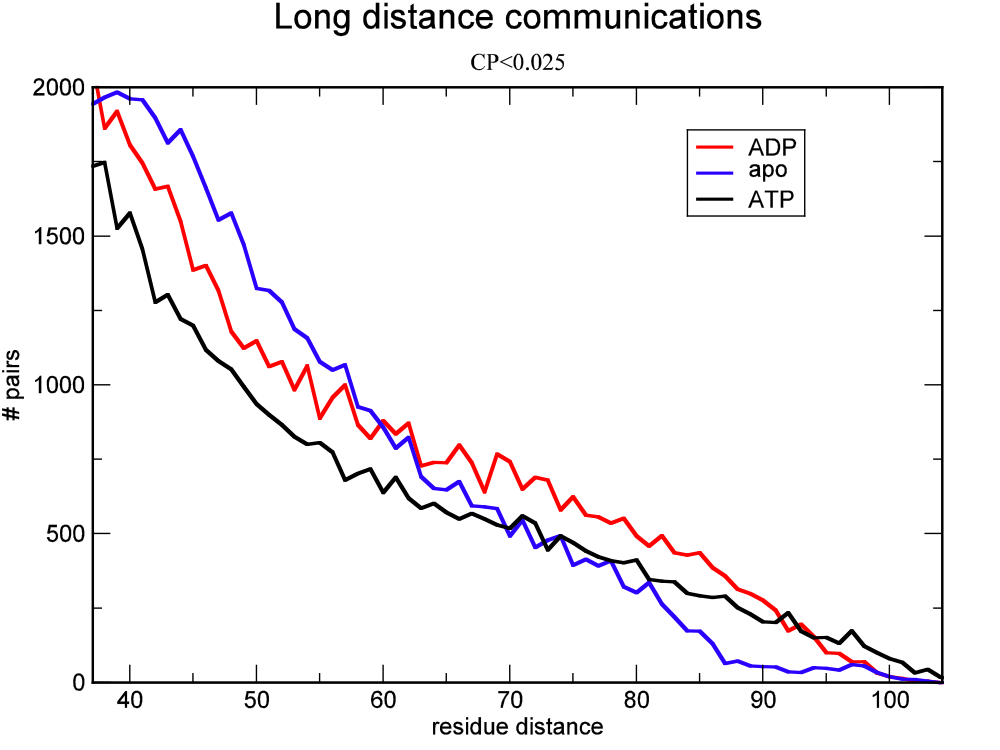

Supplement: Figure S4 — The Histogram showing the number of fast communication events (CP<0.025) for each given distance of the communicating residues. In the ATP and ADP complexes the inter-domain communications persist at extremely long range (over 80Å); while such a long distance tail is not present in the apo system. (2.25 MB TIF) [file pcbi.1000323.s004.tif]

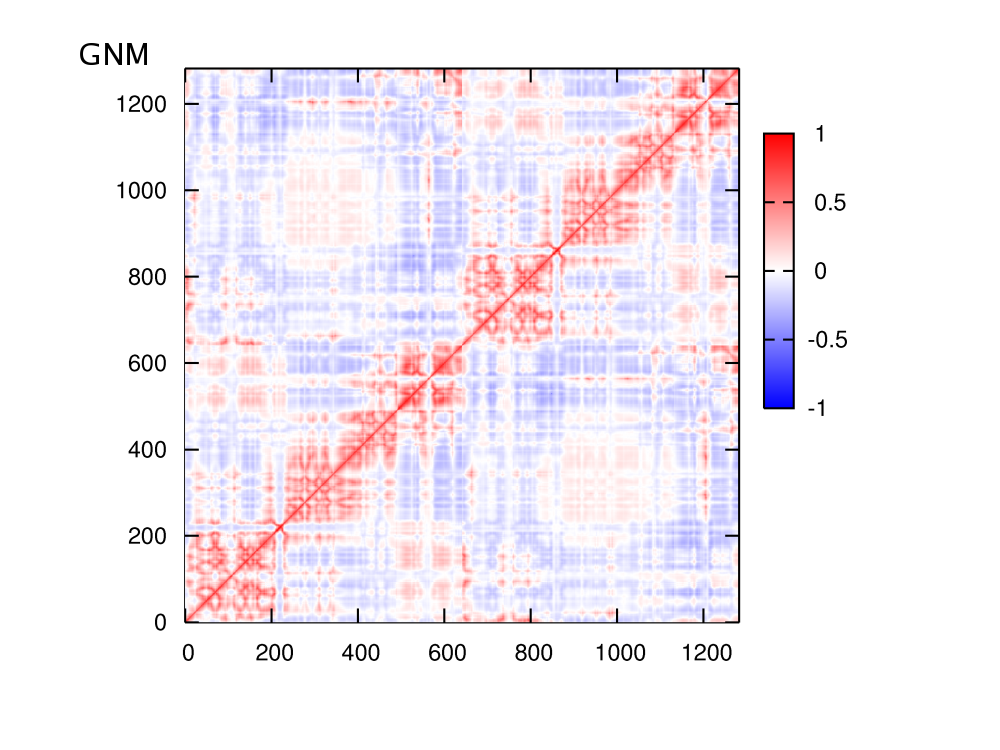

Supplement: Figure S5 — The cross-correlation matrix calculated of the Hsp90 dimer using the GNM approach. A correlation close to 1 (color code: yellow) corresponds to a highly coordinated motion of the atom pair along the same direction, whereas a negative correlation (color code: blue) indicates motion in opposite directions. (2.25 MB TIF) [file pcbi.1000323.s005.tif]

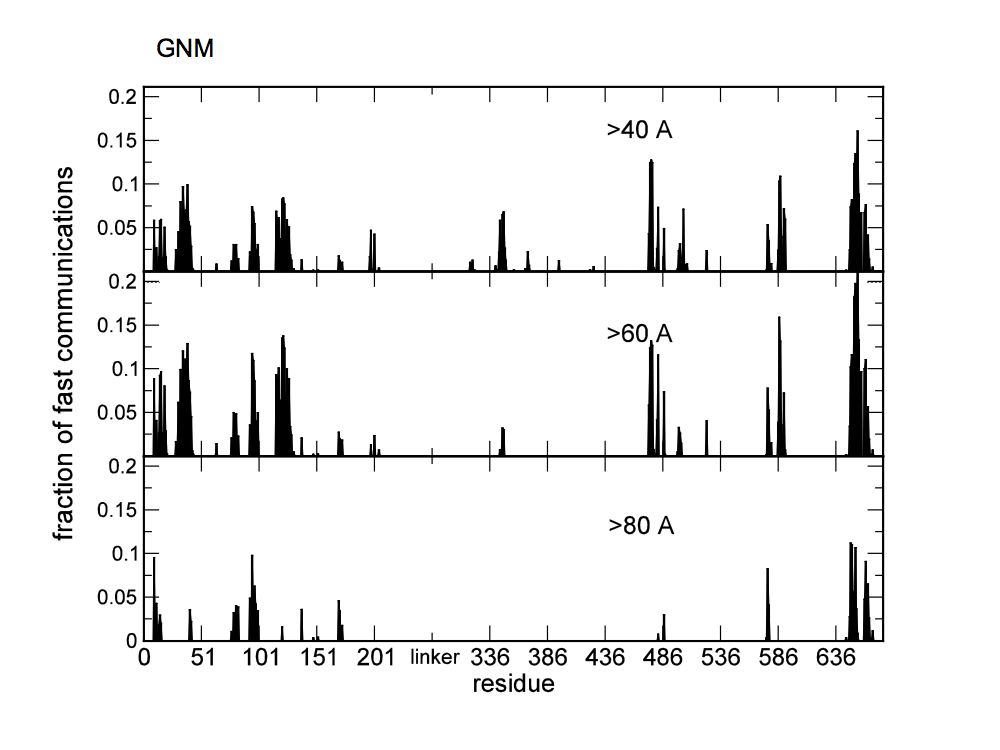

Supplement: Figure S6 — The analysis of inter-domain communication pathways in the Hsp90 dimer using the GNM approach. A Set of histograms showing the communication efficiency of all residues, evaluated at increasing distances. Each bin refers to a residue and shows the fraction of residues that are highly prone to communicate with it, i.e. such that their pair CP value is below 2.5. In each histogram only communications at distances greater than a given threshold are considered, namely over 40, over 60 and over 80 Angstroms. (2.25 MB TIF) [file pcbi.1000323.s006.tif]
